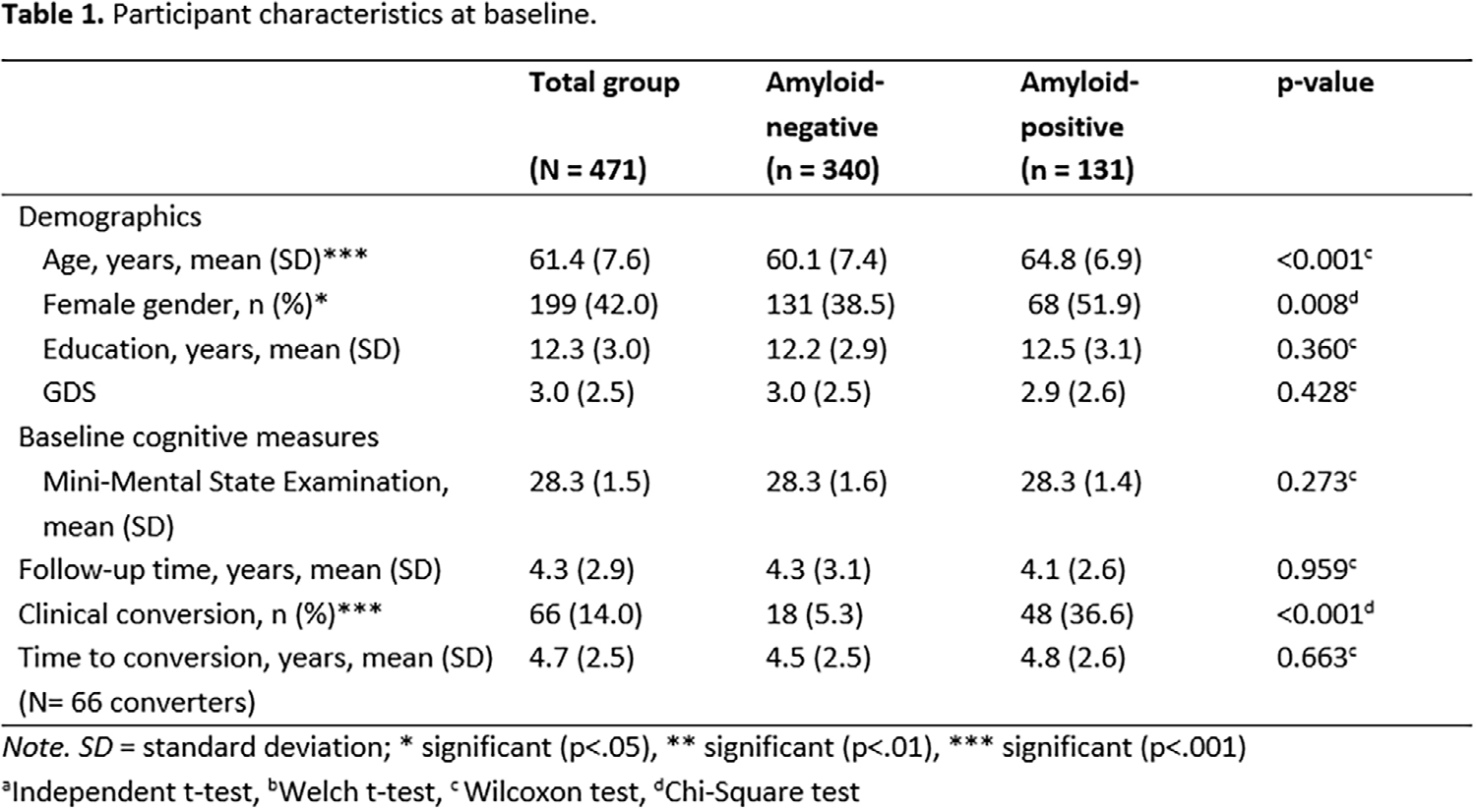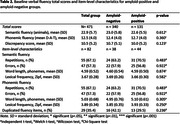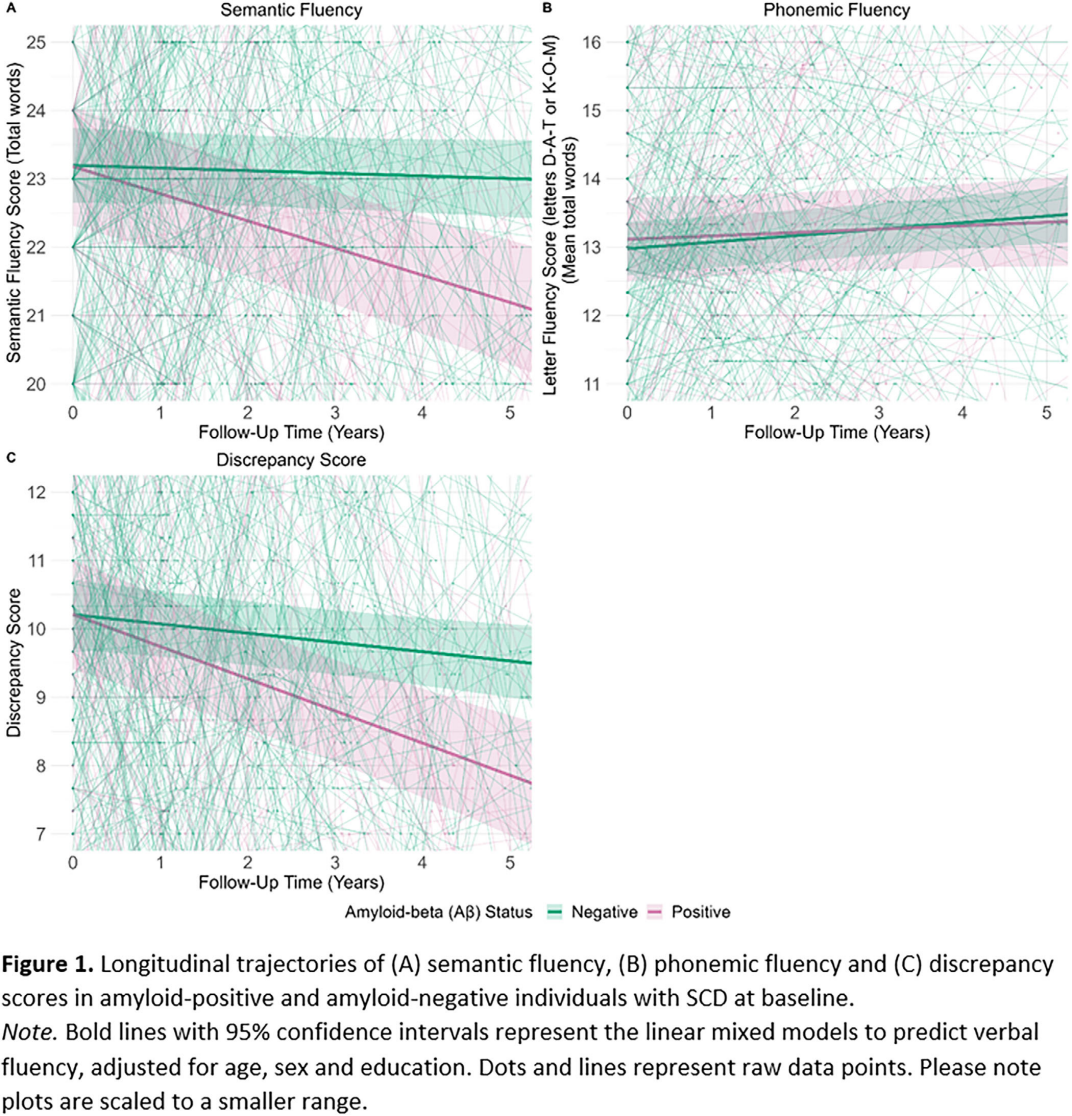# Amyloid‐positivity is characterized by decline in semantic fluency: An in‐depth investigation of verbal fluency trajectories, item‐level characteristics and its prognostic value in patients with subjective cognitive decline

**DOI:** 10.1002/alz.088963

**Published:** 2025-01-03

**Authors:** Rosanne L. van den Berg, Elke Butterbrod, Casper de Boer, Demi van de Scheur, Lisa‐Marie Schlüter, Argonde C. van Harten, Charlotte Teunissen, Elsmarieke van de Giessen, Wiesje M. van der Flier, Sietske A.M Sikkes

**Affiliations:** ^1^ Alzheimer Center Amsterdam, Neurology, Vrije Universiteit Amsterdam, Amsterdam UMC location VUmc, Amsterdam Netherlands; ^2^ Amsterdam Neuroscience, Neurodegeneration, Amsterdam Netherlands; ^3^ Faculty of Behavioural and Movement Sciences, Vrije Universiteit Amsterdam, Amsterdam Netherlands; ^4^ Neurochemistry Laboratory, Department of Clinical Chemistry, Vrije Universiteit Amsterdam, Amsterdam UMC location VUmc, Amsterdam, North Holland Netherlands; ^5^ Alzheimer Center Amsterdam, Neurology, Vrije Universiteit Amsterdam, Amsterdam UMC, Amsterdam Netherlands; ^6^ Radiology & Nuclear Medicine, Vrije Universiteit Amsterdam, Amsterdam UMC location VUmc, Amsterdam Netherlands

## Abstract

**Background:**

Verbal fluency, especially semantic fluency, may hold promise to predict clinical progression in the preclinical Alzheimer’s disease (AD) stage, where patients show no objective cognitive impairment. We examined verbal fluency trajectories in amyloid‐negative and amyloid‐positive individuals with subjective cognitive decline (SCD), as well as whether baseline fluency characteristics (total scores and item‐level) predicted progression to MCI or AD dementia.

**Method:**

We retrospectively selected data of 471 Dutch individuals with SCD, with at least 1 follow‐up fluency assessment, from the Amsterdam Dementia Cohort (Follow‐up years = 4.3±2.9, Age = 61±8, Female n = 199(42%), Amyloid‐positive n = 131(28%), clinical progression n = 66(14%), Table 1). We longitudinally measured total scores for semantic (animals) and phonemic fluency (alternate versions) and semantic‐phonemic‐discrepancy scores (subtracting phonemic from semantic fluency). For a subset (n = 82) we obtained baseline item‐level characteristics: lexical frequency and word length (both continuous), errors, repetitions and duplications between semantic and phonemic fluency (all dichotomized). We compared baseline fluency measures between amyloid groups and clinical progression groups. Fluency trajectories and their associations with amyloid‐status were examined with linear mixed models. We examined associations between baseline fluency and progression to MCI/AD dementia using Cox proportional hazard models. Cox models for total and discrepancy scores were performed in the total group including a fluency*amyloid interaction, whereas models for item‐level metrics only included amyloid‐positive individuals (n = 44). All models were adjusted for age, sex and education.

**Results:**

At baseline no significant differences in any of the fluency measures between amyloid groups were observed (*p’s* > 0.05, Table 2). Longitudinally, amyloid‐positive individuals declined more steeply on semantic fluency (*β* = ‐0.37,*95%CI* = ‐0.53—0.20) and phonemic‐semantic‐discrepancy (*β* = ‐0.32,*95%CI* = ‐0.49—0.14) compared to amyloid‐negative individuals. Groups did not differ on phonemic fluency (*β* = ‐0.05*,95%CI* = *‐*0.15–0.05, Figure 1). While baseline discrepancy scores were lower in patients who later progressed (Mean = 9.0±SD = 5.4) than in non‐progressors (Mean = 10.7±SD = 5.1,*W* = 14317,*p* = 0.023), none of the baseline fluency measures predicted clinical progression after adjusting for covariates (*p’*s>0.05).

**Conclusion:**

Longitudinal decline in semantic fluency and discrepancy scores, but not phonemic fluency, was associated with amyloid positivity in individuals with SCD. Baseline fluency did not predict clinical progression. These findings suggest that longitudinal follow‐up is essential to capture semantic fluency decline that is associated with AD pathophysiology.